# Proteomic changes of the porcine small intestine in response to chronic heat stress

**DOI:** 10.1530/JME-15-0161

**Published:** 2015-12

**Authors:** Yanjun Cui, Xianhong Gu

**Affiliations:** 1 State Key Laboratory of Animal Nutrition, Institute of Animal Sciences, Chinese Academy of Agricultural Science, Beijing, 100193, People's Republic of China

**Keywords:** heat stress, intestine function, morphology, proteomic, pig (*Sus scrofa*)

## Abstract

Acute heat stress (HS) negatively affects intestinal integrity and barrier function. In contrast, chronic mild HS poses a distinct challenge to animals. Therefore, this study integrates biochemical, histological and proteomic approaches to investigate the effects of chronic HS on the intestine in finishing pigs. Castrated male crossbreeds (79.00±1.50 kg BW) were subjected to either thermal neutral (TN, 21 °C; 55%±5% humidity; *n*=8) or HS conditions (30 °C; 55%±5% humidity; *n*=8) for 3 weeks. The pigs were sacrificed after 3 weeks of high environmental exposure and the plasma hormones, the intestinal morphology, integrity, and protein profiles of the jejunum mucosa were determined. Chronic HS reduced the free triiodothyronine (FT_3_) and GH levels. HS damaged intestinal morphology, increased plasma d-lactate concentrations and decreased alkaline phosphatase activity of intestinal mucosa. Proteome analysis of the jejunum mucosa was conducted by 2D gel electrophoresis and mass spectrometry. Fifty-three intestinal proteins were found to be differentially abundant, 18 of which were related to cell structure and motility, and their changes in abundance could comprise intestinal integrity and function. The down-regulation of proteins involved in tricarboxylic acid cycle (TCA cycle), electron transport chain (ETC), and oxidative phosphorylation suggested that chronic HS impaired energy metabolism and thus induced oxidative stress. Moreover, the changes of ten proteins in abundance related to stress response and defense indicated pigs mediated long-term heat exposure and counteracted its negative effects of heat exposure. These findings have important implications for understanding the effect of chronic HS on intestines.

## Introduction

Increasing global warming has resulted in increased research on the detrimental effects of heat stress (HS) on animal welfare and livestock production (Laganá *et al*. 2007, Yu *et al*. 2010). Pigs experience HS when ambient temperatures exceed their thermal neutral (TN) zone (16–22 °C for growing-finishing pigs) (Coffey *et al.* 1995). Compared with other species, finishing pigs are more susceptible to HS owing to their high metabolic heat production, quick fat deposition and lack of sweat glands (Dallaire *et al*. 1996). HS in pigs reduces food intake, body weight (BW) gain and meat quality, all of which potentially cause large economic losses (Pearce *et al*. 2012, Pearce *et al*. 2013*a*, Cruzen *et al*. 2015). For instance, HS has been estimated to cost the US swine industry losses of more than $300 million annually (St-Pierre *et al*. 2003).

The gastrointestinal tract performs the critical function of selectively absorbing nutrients and water (Pearce *et al*. 2014*b*), and acts as a defensive barrier against endogenous and dietary pathogens as well as toxic compounds (Hirata *et al*. 2007). Gastrointestinal changes in these functions and integrity could be detrimental to health, performance and welfare of mammals. Therefore, intestinal health is of great importance in both human medicine and animal production. The gastrointestinal tract is highly sensitive to heat loads (Kregel 2002). Previous studies have shown that acute HS compromises the pig gastrointestinal tract epithelium and increases intestinal permeability to endotoxins such as lipopolysaccharide (LPS) (Pearce *et al*. 2013*b*), leading to low animal yield and performance, and increased morbidity and mortality (Liu *et al*. 2009). Moreover, investigations have been focused on mechanisms underlying the effect of acute HS on intestinal function and integrity. Acute HS causes hypoxia and inflammation of the intestinal epithelium (Lambert 2009, Qi *et al*. 2011), both of which regulate intestinal tight junction (TJ) proteins including the myosin light chains (MLC), occluding, claudin, and MLC kinase (MLCK) (Turner 2006, Pearce *et al*. 2013*b*), which maintain intestinal function and integrity. In addition, HS reduces the thyroid hormone (triiodothyronine (T_3_)) level (Silva 2003), leading to the down-regulation of intestinal alkaline phosphatase (AP) in transcriptional level and thus influencing intestinal function (Hodin *et al*. 1992). Acute HS also affects the intestine at the molecular level, including changes in gene and protein expression, and biochemical adaptations. For example, the investigation reported by Yu *et al*. (2010) demonstrated that acute HS could increase HSP27, HSP70 and HSP90 expression and trigger MAPK signaling pathways in the pig jejunum after 3 days of HS. Another similar study demonstrated that acute HS resulted in increased HSP 70 mRNA and protein abundance (Pearce *et al*. 2014*a*).

Interestingly, the majority of these studies have analyzed acute HS impact on the performance, physiology and molecular response of pig. In contrast to acute HS (40–42 °C, less than 24 h), chronic HS (33–35 °C, more than 24 h) poses a distinct challenge to animals. Compared to hyperthermia and even death caused by acute HS, chronic HS can be tolerated for a longer period of time (weeks) (Horowitz, 2002). Nevertheless, the phenotypic changes that were reported in response to chronic HS in a variety of species including finishing pigs (Gordon 1993, Hao *et al*. 2014) suggest that chronic mild HS alters animal performances and physiology. Response to HS is a complex biological process that involves many proteins. In contrast to conventional biochemical approaches that address one or a few specific proteins at a time, proteomics technologies facilitate the analysis of thousands of proteins, offering powerful tools for comprehensively assessing molecular alterations of intestines due to heat exposure. Molecular mechanisms underlying the effects of chronic HS in the intestine of finishing pigs have not been extensively studied. We hypothesized that changes of intestinal proteome would mediate the effects of HS on intestinal integrity, function and metabolism in finishing pigs.

Thus, this study aimed to investigate the proteomic response of the small intestine to chronic HS in finishing pigs and to identify novel intestinal protein profiles that could explain how pigs mediate and manage long-term heat exposure.

## Materials and methods

### Animals and experimental design

Sixteen castrated male DLY (crossbreeds of Landrace×Yorkshire sows and Duroc boars) pigs were randomly selected from 8 litters from a pig breeding farm (Beijing, China), and were transported to the State Key Laboratory of Animal Nutrition (Beijing, China). Individual pig BWs were 79.00±1.50 kg. Pigs were randomly allocated to either the control or heat-treated group (eight pigs per treatment group). The initial BW and litter origin of all pigs in each group was noted. Four pigs from one group were housed in an artificial climate chamber (2.1×4.8 m^2^, luminance 100 l×, photoperiod 14 h light, humidity 55%±5%), with four artificial climate chambers being used. All animals were fed with standard feed according to the NRC (1998) recommendations. The feed contained no antibiotics (Table 1).

Before the experiment, the animals were allowed to acclimate to the artificial climate chamber at 22 °C for 7 days. Sixteen pigs were then randomly assigned to the two treatments. One group of eight pigs was housed in TN conditions (22 °C) with *ad libitum* feed intake. The remaining eight pigs were subjected to HS (30 °C) with *ad libitum* feed intake. To minimize damage caused by acute high temperature (30 °C) in the HS group, the artificial temperature climate of the chamber was gradually increased and kept at 27 °C on d1, and then raised to 28 °C on d2. Thereafter, the temperature was kept constant at 30 °C, while control animals were maintained at 22 °C, until the end of the experiment. The experimental period lasted for 3 weeks.

The study was conducted at the State Key Laboratory of Animal Nutrition. The experiment was performed in accordance with guidelines of the Beijing Animal Ethics Committee and received prior approval from the Chinese Academy of Agricultural Sciences Animal Care and Use Committee.

### Blood and tissue collection

Prior to sacrifice, venous blood was immediately collected from the jugular vein using venipuncture and centrifuged at 1500 ***g***at 4 °C for 10 min to obtain K_2_EDTA plasma. The plasma were subsequently transferred into 1.5 ml sterile tubes and stored at −20 °C until later assay. Thereafter, the pigs were slaughtered using a head-only electric stun tong apparatus (Xingye Butchery Machinery Co. Ltd, Changde, Hunan Province, China).

Intestinal tissues were obtained immediately following exsanguination euthanasia. The intestinal sections were quickly divided into the duodenum (5 cm from the pylorus), and jejunum (150 cm anterior to the ileocecal valve) and the ileum (150 cm proximal from the ileal-cecal junction). The jejunum was selected and thoroughly rinsed with physiological saline then cut into 1 cm length segments. It was fixed in 10% neutral formalin then used for histological analysis. Mucosa from the remaining segment was obtained as described previously (Wang *et al*. 2007) and snap frozen in liquid nitrogen then stored at −80 °C until biochemical and molecular analyses. Jejunum tissue from the small intestine was selected for analysis, as this tissue is important for nutrient absorption, high blood flow, and sensitivity to hypoxia and inflammation (Maier *et al*. 2009).

### Quantification for plasma thyroid hormones and growth hormone

Plasma T_3_, free T_3_ (FT_3_), thyroxine (T_4_), free thyroxine (FT_4_), and growth hormone (GH) were quantified by RIA, using standard RIA kits (Huaying Bio-Tech Research Institute, Beijing, China). The intra-assay coefficient of variation was <5%. Plasma levels of T_3_, FT_3_, T_4_ and GH are expressed as ng/ml of serum, FT_4_ as pg/ml.

### Intestinal morphology assessment

The intestinal samples fixed in formalin were sent to the State Key Laboratory of Animal Nutrition, Institute of Animal Sciences. Jejunum sections were embedded in paraffin and transversely sectioned in (5 μm thick) and stained with hematoxylin and eosin following deparaffinization and dehydration. Intestinal tissues and structures were observed using a BH2 Olympus microscope (Olympus, Tokyo, Japan) and analyzed using an image analysis system (Olympus 6.0). Villi height, crypt depth and their ratios were assessed following the method of Gabler *et al*. (2007).

### Intestinal integrity and function assay

Plasma d-lactate concentration was measured using a porcine-specific ELISA according to the manufacturer's instructions (Beijing Chemclin Biotech Co., Ltd, Beijing, China). The intra-assay coefficient of variation was <5%. Plasma d-lactate level is expressed as μg/ml of plasma.

AP activity of jejunum mucosal was determined by a kinetic based assay using a commercially available kit (Nanjing Jiancheng Co., Ltd, Nanjing, China). Protein was extracted from the jejunum mucosal and protein concentration was determined using bovine serum albumin (fraction V) as the protein standard. AP activity of jejunum mucosal are expressed as μ/g protein. The intra-assay coefficient of variation was <5%.

### Proteomic analysis

#### Jejunum mucosa protein extraction

Total proteins were extracted from jejunum mucosal scrapings by following an existing procedure with slight modifications (Wang *et al*. 2009). In brief, frozen samples of jejunum mucosal scrapings from all pigs in two groups were crushed in a mortar containing liquid nitrogen. The powder (approximately 100 mg per sample) was transferred to sterile tubes with lysis buffer (LB; containing 7 M urea, 2 M thiourea, 4% w/v CHAPS, 1% w/v DTT, 1% v/v IPG Buffer pH 4–7, 1% v/v proteinase inhibitor cocktail). The mixture was sonicated in an ice bath using a Model VCX 500 Ultrasonicater (Sonics & Materials, Newtown, CT, USA) at 20% power output for 10 min with 2-s on and 4-s off cycles. Subsequently, the lysed cell suspension was incubated at room temperature for 1 h to solubilize proteins. After centrifugation at 40 000 ***g*** and 4 °C for 40 min, the supernatant protein was collected and its protein concentration was determined according to a modified Bradford assay (Ramagli & Rodriguez 1985). The protein concentration was 6.84±0.42 mg/ml.

#### 2D gel electrophoresis

A 1 mg protein sample was loaded on a 24 cm IPG strip (immobilized pH gradient, pH 4–7, linear, GE Healthcare) (Amersham Bioscience, Uppsala, Sweden). Each protein sample was assessed in triplicate. Isoelectric focusing (IEF) was carried out at 20 °C for 14 h at 30 V, 2 h at 200 V, 0.5 h at 500 V, 1 h at 1000 V, 3 h at 8000 V, and then held at 8000 V until a total of at least 60 000 Vh was reached (Ettan IPGphorII, GE Healthcare, Uppsala, Sweden). Focused IPG strips were equilibrated for 15 min in equilibration buffer (6 M urea, 30% glycerol, 2% SDS, 50 mM Tris pH 8.8, 1% DTT) under gentle agitation, and then for an additional 15 min in the same buffer, except that DTT was substituted with 2.5% iodoacetamide. After equilibration, the strips were transferred to vertical slab gels (12% SDS–PAGE) for second-dimensional electrophoresis with the Ettan DALT six gel system (GE Healthcare).

#### Image acquisition and analysis

Gels were fixed for about 8 h in a solution containing (10% (v/v) acetic acid, 40% (v/v) ethanol, and 50% (v/v) water), washed three times in water, and then stained with Coomassie colloidal blue G-250 according to the GE handbook (GE Healthcare) with minor modifications. Gel images were acquired with the PowerLook 2100XL color scanner (UMAX Technologies, Atlanta, CA, USA) at a resolution of 16 bits and 300 dpi, and were assayed by Image master 2D Platinum Software Version 6.0 (GE Healthcare). To limit experimental variation among 2D gels, quantitative comparison of protein spots was performed on the base of their percentage volumes. All automatic spot detections for each gel were manually inspected and edited as necessary to confirm the absence of mismatched and unmatched spots. One-way ANOVA and comparison of treatment means were carried out on the SAS program. Differentially expressed protein spots were (1) consistently present in all replicates and (2) changed abundance by at least ±1.2-fold, with an error probability of *P*≤0.05.

### Protein identification

The MALDI-TOF-MS/MS analysis was based on the method previously described (Xiong *et al*. 2011). Selected spots were excised from the gels and destained using a 20% w/v sodium thiosulfate and 1% w/v potassium ferricyanide for 5 min. The supernatant was removed and the gel spots were washed twice with 25 mM ammonium bicarbonate in 50% v/v ACN for 20 min. The gel spots were then washed in ACN, dried in a Speed-Vac and digested with 20 mg/ml of trypsin in 25 mM ammonium bicarbonate for 12 h at 37 °C. Tryptic peptides were passed through C18 Zip-Tips and mixed with 5 mg/ml of an R-cyano-4-hydroxycinnamic acid as matrix and subjected to MALDI-TOF/TOF analysis (4700 Proteomics Analyzer, Applied Biosystems). For database searching, data files obtained from MALDI-TOF/TOF mass spectra were submitted to the MASCOT search engine using Daemon 2.1.0 (Matrix Science; http://www.matrixscience.com) on a MASCOT server version 2.2.1. The data were searched against the NCBInr database. The peptides were constrained to be tryptic with a maximum of one missed cleavage. Carbamidomethylation of cysteine was considered a fixed modification, and oxidation of methionine residues was considered as a variable modification. Protein identifications were accepted if they established a probability >95% and contained at least two identified peptides having maximal peptide coverage.

### Bioinformatic approach

To enrich the differentially expressed proteins with respect to specific functional terms, the protein lists were analyzed using the plug-in of the Cytoscape software: ClueGO (http://www.ici.upmc.fr/cluego/) (Saito *et al*. 2012) with the Gene Ontology database (release date: June 2014). The ontology selection on the base of biological processes and enrichment analysis was performed by the right-side hyper-geometric statistic test and its probability value was corrected by the Bonferroni's method (Bindea *et al*. 2009). A pathway enrichment analysis of the differentially expressed proteins (Ashburner *et al*. 2000) was conducted using ClueGO software and applying database from the Kyoto Encyclopedia of Genes and Genomes (KEGG) database (release date: March 2014).

A protein interaction network of the differentially regulated proteins was analyzed using the online database resource Search Tool for the Retrieval of Interacting Genes (STRING 9.1) (Szklarczyk *et al*. 2011). The protein regulation networks and protein interaction maps are in the *Sus scrofa* molecular networks database. The network nodes are the proteins, and the edges represent the predicted functional associations. An edge may be drawn with up to seven differently colored lines. These lines represent the existence of the seven types of evidence used for predicting the associations. The interactions between the imported proteins and all proteins stored in the database were then identified.

### Validation of differentially expressed proteins by Western blot

Western-blotting analysis was used to validate the main differentially expressed proteins. Total protein (30 μg/sample) was separated by electrophoresis (Bio-Rad) on 10% SDS–PAGE, and transferred to a PVDF membrane (Millipore, Billerica, MA, USA). The blotted membrane was blocked for 2 h at room temperature in 1× TBST (0.05% Tween 20, 100 mM Tris–HCl and 150 mM NaCl (pH 7.5)) containing 5% fat-free dry milk, and then incubated under gentle agitation overnight at room temperature in the presence of the primary antibodies: heat shock protein 105 kDa (HSPH1; spot 6), 1:5000 dilution of purified mouse monoclonal anti-HSPH1 antibody (Abcam, AB109624 Cambridge, MA, USA); heat shock 70 kDa protein 1B (HSPA1A; spot 7), 1:5000 dilution of purified mouse monoclonal anti-HSPA1A antibody (TDY062F, Beijing Biosynthesis Biotechnology Co., Ltd, Beijing, China); glyceraldehyde-3-phophate dehydrogenase (GAPDH), 1:2000 dilution of purified mouse monoclonal anti-GAPDH antibody (TDY062, Beijing Biosynthesis Biotechnology Co., Ltd); hsp27(HSPB1; spot 86), 1:1000 dilution of purified rabbit polyclonal anti-HSPB1 protein antibody (Abcam, AB2790), which could bind to their specific protein. The blots were extensively washed with TBST buffer for 10 min×3 times and incubated under gentle agitation with the secondary antibodies for immunodetection. The antigen-antibody interaction was carried out for 1 h, and the cross-reacting proteins were detected using ECL (Perkin Elmer Life Sciences, Boston, MA, USA). The protein bands were visualized with a chemiluminescence substrate using a gel-imaging system (Tanon Science and Technology, Shanghai, China) with Image Analysis Software (National Institutes of Health, Bethesda, MD, USA). In all instances, density values of bands were corrected by subtraction of the background values. GAPDH was used as the internal reference protein. Bands were standardized to the density of GAPDH and normalized fold expression represented as a ratio of each protein to GAPDH.

### Statistical analysis

Statistical analyses were conducted using SAS version 8.2 software (SAS Institute, Cary, NC, USA). Data were expressed as mean±s.d. The Student's *t*-test was used for statistical analysis and a difference at *P*≤0.05 was considered statistically significant.

## Results

### Effect of chronic HS on plasma hormone

The comparison of levels in heat exposure pigs and control are shown in Table 2. As compared to the control group, the levels of plasma FT_3_ and GH were significantly decreased (*P*=0.008 and* P*=0.015 respectively), while the level of T_3_, T_4_ and FT_4_ were not changed (*P*>0.05).

### Effect of chronic HS on jejunum morphology, integrity and function

**Figure 1:**
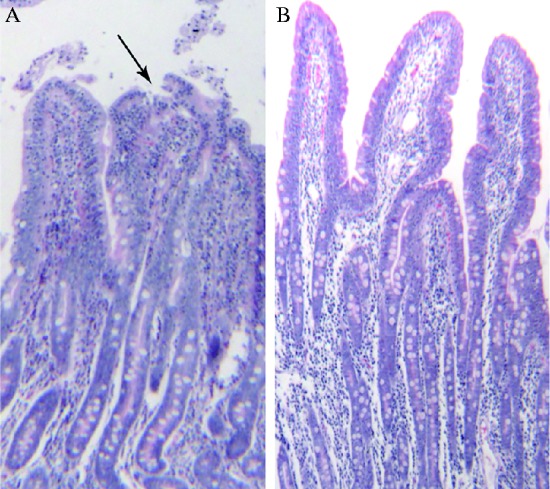
Photomicrographs of hematoxylin- and eosin-stained sections of the pig small intestine from heat treated and control animals after 3 weeks of treatment (200× magnification). (A and B) Heat treated jejunums and control respectively. Damage to the intestinal villi is obvious, with desquamation at the tips of the intestinal villi. Abnormal microstructures are indicated with arrowheads.

Chronic HS resulted in morphological alterations of the porcine small intestine. Desquamation was prevalent at the tips of the intestinal villus in heat-stressed pigs as shown in Fig. 1. Compared with the TN group, HS reduced villus height and crypt depth (*P *≤ 0.05, Table 3). There were no differences detected between TN and HS in terms of villus: crypt ratio (*P*>0.05, Table 3).

Plasma d-lactate concentrations were measured as a marker of intestine integrity. We demonstrated that concentrations of plasma d-lactate were increased 19.5% due to HS (*P*≤0.05). As expected, HS resulted in a 33% decrease in AP activity of jejunum mucosa (*P*<0.05, Table 4), a measure of intestine function.

### Proteomic alterations of jejunum mucosa in response to HS

**Figure 2:**
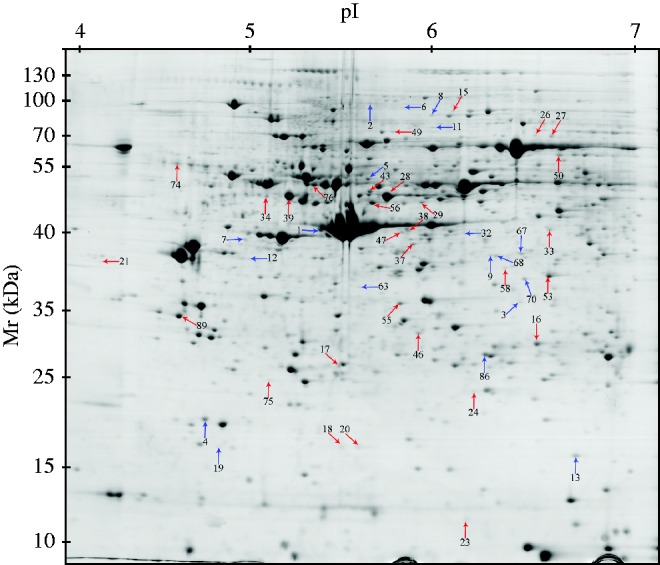
Protein profile patterns in the jejunum mucosa of finishing pigs. Protein spots showing significant differences (1.3-fold, *P*≤0.05) were cut out and identified by MALDI-TOF/TOF MS. Protein spots of differential abundance with known identities are marked with color arrows, blue indicates up-regulated and red indicates down-regulated.

**Figure 3:**
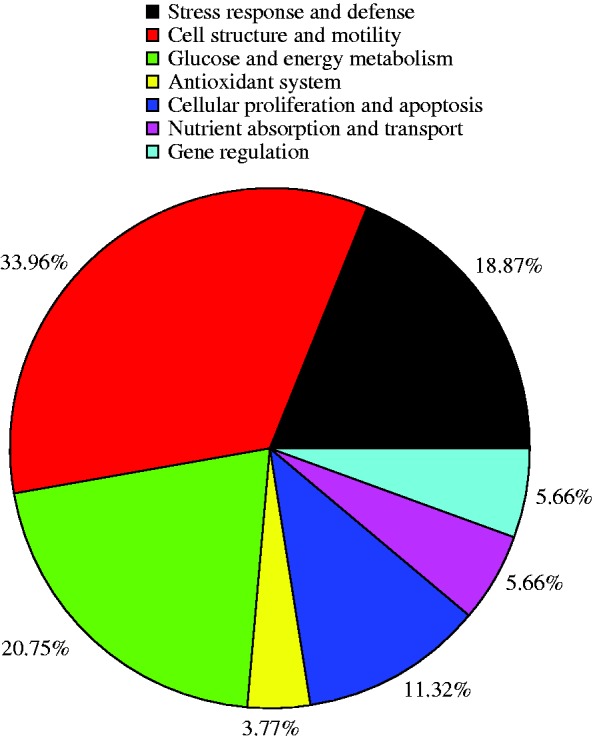
Functional classification of the differentially expressed proteins identified from the jejunum mucosa of finishing pigs. The color codes represent different protein functional groups.

**Figure 4:**
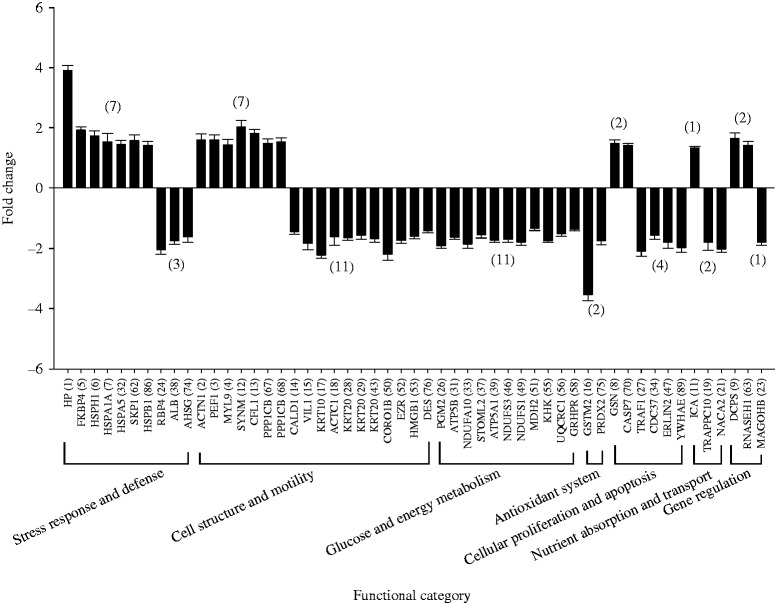
Quantitative analysis of the proteins of differential abundance from the jejunum mucosa of finishing pigs.

Finishing pigs were subjected to chronic HS (30 °C) and jejunum mucosa changes in the protein profiles were determined by a 2DE approach. A total of 992 protein spots were detected on 2D gels of jejunum mucosa and spot localization on the map is shown in Fig. 2. There were 53 differentially expressed proteins and their biochemical information is summarized in Table 5. Based on their biological functions, these proteins were classified into seven groups: i) stress response and defense system (18.87%), ii) cell structure and motility (33.96%), iii) glucose and energy metabolism (20.75%), iv) antioxidant system (3.77%), v) cellular apoptosis (11.32%) vi) nutrient absorption and transport (5.66%), and vii) gene regulation (5.66%) (Fig. 3). Those related to cell structure and motility, glucose and energy metabolism, stress response and defense were predominant and accounted for approximately 73% of the differential proteins. A comparison of differentially expressed proteins between the groups showed that fewer protein species were up-regulated in pigs subjected to chronic HS (19 vs 34) (Fig. 4). These 19 up-regulated protein species were distributed in five categories: seven in stress response and defense, seven in cell structure and motility, one in nutrient absorption and transport, two in cellular proliferation and apoptosis, and two in gene regulation. The 34 down-regulated protein species were distributed in seven categories: three in stress response and defense, 11 in cell structure and motility, 11 in glucose and energy metabolism, two in the antioxidant system, four in cellular proliferation and apoptosis, another two in nutrient absorption and transport, and one in gene regulation.

### Confirmation of differential proteins in abundance by Western blot

**Figure 5:**
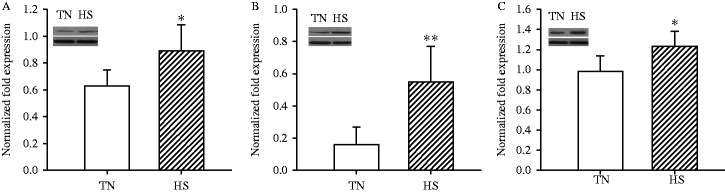
Western blotting analysis of jejunum mucosa proteins, HSPH1 (A), HSPB1 (B), and HSPA1A (C). Data are mean±s.d., *n*=8 pigs for each group. *P<0.05 and **P<0.01 before vs after heat stress.

Immunoblotting was further performed to verify the proteomic results. Confirmation of the three stress-response marker proteins HSPH1 (spot 6), HSPA1A (spot 7) and HSPB1 (spot 86) was carried out using antibodies. The results of the immunoblotting analysis were consistent with the 2DE results (Fig. 5).

### Bioinformatics analysis of differentially expressed proteins

**Figure 6:**
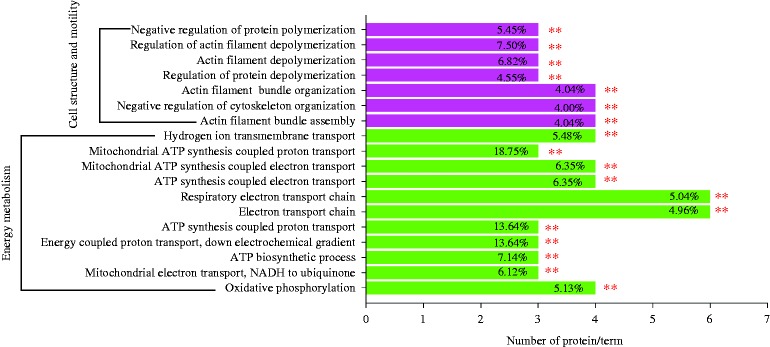
Functional enrichment analysis of the proteins of differential abundance from the jejunum mucosa of finishing pigs using the ClueGO software. **P<0.01.

Gene Ontology (GO) enrichment analysis and functional annotation are useful for the analysis of large proteomic and genomic datasets. Significantly overrepresented GO terms were examined to determine the putative biological events behind the data and provide a primary overview of the jejunum mucosa proteome. Functional enrichment analysis of all differential proteins was conducted using the ClueGo software. The result showed that two major functional groups were significantly enriched: cell structure and motility and energy metabolism (Fig. 6). Proteins that were enriched in the cell structure and motility included alpha-actinin-1 (ACTN1; spot 2), villin 1 (VIL1; spot 15), cofilin-1 (CFL1; spot 13), coronin-1B (CORO1B; spot 50) and ezrin (EZR; spot 52). NADH dehydrogenase (ubiquinone) 1 alpha subcomplex subunit 10 (NDUFA10; spot 33), NADH-coenzyme Q reductase (NDUFS3; spot 46), NADH-ubiquinone oxidoreductase 75 kDa subunit (NDUFS1; spot 49), cytochrome b-c1 complex subunit 1, mitochondrial (UQCRC1; spot 56), stomatin-like protein 2, mitochondrial (STOML2; spot 37), ATP synthase subunit alpha, mitochondrial (ATP5A1; spot 39) and ATP synthase subunit beta, mitochondrial (ATP5B; spot 31) were significantly enriched in the energy metabolism.

KEGG pathway enrichment analysis of the differentially expressed proteins revealed protein functional information in the metabolic pathway. The pathway analysis showed that 11 differential proteins were significantly enriched in the two pathways that were involved in multiple biological processes containing oxidative phosphorylation and regulation of actin cytoskeleton (Table 6).

**Figure 7:**
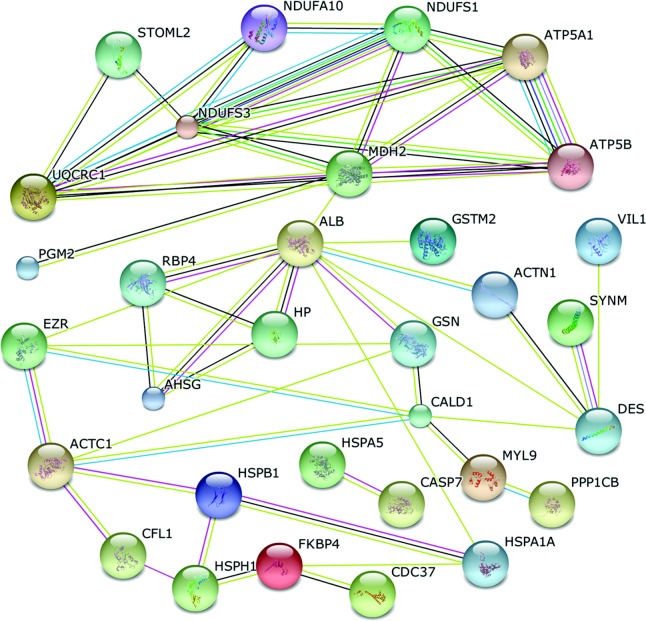
Biological interaction network of the identified differentially expressed proteins from the jejunum mucosa of finishing pigs. A red line, fusion evidence; a green line, neighborhood evidence; a blue line, co-occurrence evidence; a purple line, experimental evidence; a yellow line, text mining evidence; a light blue line, database evidence; and a black line, coexpression evidence.

Proteins function as elementary parts of protein complexes in living cells. However, they do not act independently. Accordingly, 32 proteins were recognized as key nodes with various relationships in protein–protein interactions (PPI) (Fig. 7). Using the online tools of STRING 9.1, we demonstrated that 11 proteins were related to cell structure and motility: alpha-actinin-1 (ACTN1; spot 2), myosin regulatory light chain, LC20 (MYL9; spot 4), cardiac muscle alpha actin 1 (ACTC1; spot 18), villin 1 (VIL1; spot 15), desmin (DES; spot 76), cofilin-1 (CFL1; spot 13), peflin (PEF1; spot 3), synemin (SYNM; spot 12), non-muscle caldesmon (CALD1; spot 14), ezrin (EZR; spot 52), and serine/threonine-protein phosphatase PP1-beta catalytic subunit (PPP1CB; spot 67 and 68). The second most represented group included proteins related to stress response and defense, and antioxidant systems: peptidyl-prolyl cis-trans isomerase (FKBP4; spot 5), heat shock protein 105 kDa (HSPH1; spot 6), hsp27 (HSPB1; spot 86), heat shock 70 kDa protein 5 (HSPA5; spot 32), heat shock 70 kDa protein 1B (HSPA1A; spot 7), haptoglobin (HP; spot 1), retinol-binding protein 4 (RBP4; spot 24), serum albumin (ALB; spot 38), alpha-2-HS-glycoprotein (AHSG; spot 74), and glutathione S-transferase mu 2 (GSTM2; spot 16). In contrast, proteins involved in glucose and energy metabolism ranked as the third most represented, each linked to the network through nine proteins: phosphoglucomutase 2 (PGM2; spot 26), Malate dehydrogenase (MDH2; spot 51), NADH-coenzyme Q reductase (NDUFS3; spot 46), NADH-ubiquinone oxidoreductase 75 kDa subunit (NDUFS1; spot 49), cytochrome b-c1 complex subunit 1, mitochondrial (UQCRC1; spot 56), stomatin-like protein 2, mitochondrial (STOML2; spot 37), ATP synthase subunit alpha, mitochondrial (ATP5A1; spot 39) and ATP synthase subunit beta, mitochondrial (ATP5B; spot 31). In addition, three proteins were involved in cellular apoptosis: gelsolin (GSN; spot 8), CDC37 cell division cycle 37 protein (CDC37; spot 34), and Caspase-7 (CASP7; spot 70).

## Discussion

Our previous experiment (Hao *et al*. 2014) demonstrated that in finishing pigs chronic mild HS (30 °C for 3 weeks) resulted in decreased feed intake, daily BW gain and increased rectal temperature, respiration rate and plasma cortisol. The alterations in these parameters, commonly considered indicators of the consequences of HS on animal physiology (Morera *et al*. 2012), indicated that our finishing pigs were under conditions of moderate hyperthermia. In order to survive in a high temperature environment, animals have developed specific responses to hyperthermia by regulating the endocrine systems. We observed that chronic HS lowered FT_3_ and GH in heat-stressed pigs. This finding was in line with the results of similar HS experiments in Holstein cows and cattle (Mohammed & Johnson 1985, Pereira *et al*. 2008). The decline in thyroid hormones along with decreased plasma growth hormone (GH) level has a synergistic effect to reduce heat production.

HS leads to increased intestinal permeability in various mammalian species (Barthe *et al*. 1998, Lambert *et al*. 2002), facilitating the passive non-mediated diffusion of both small (e.g. d-lactate) and large molecules (e.g. lipopolysaccharide, LPS) from the gastrointestinal lumen to the blood. In the current experiment blood d-lactate concentration, a product of microbial metabolism, was measured as a biomarker of leaky guts (Nielsen *et al*. 2012, Sanz Fernandez *et al*. 2014). As expected, d-lactate increased during HS, suggesting that the intestinal barrier function was compromised. In support of this, intestinal AP reduced due to chronic HS. In fact, intestinal AP plays important roles in LPS dephosphorylation, reduction of LPS-induced intestinal inflammation and restriction of bacterial translocation (Goldberg *et al*. 2008). Therefore, decrease in intestinal AP activity as a result of HS reduces intestinal capacity to detoxify LPS which in turn may lead to intestinal inflammation. Furthermore, HS decreased the thyroid hormone (T_3_) level (Silva 2003) in the current study, which may result in the down-regulation of IAP in transcriptional level (Hodin *et al*. 1992), thus influencing intestinal barrier function.

Morphological changes in the small intestine were also observed. Desquamation of the mucosal epithelium and shortened height of intestinal villi and crypts indicates damage to the intestinal epithelium. This could lead to increased permeability (Lambert *et al*. 2002).

Therefore, chronic HS can reduce intestinal integrity and function. Herein, we extended our analysis to the proteomic response of porcine small intestines to chronic HS. Proteomics identified 53 proteins in small intestine that were affected by HS. These proteins are involved in stress response and defense, cell structure and motility, glucose and energy metabolism, antioxidant, cellular apoptosis, nutrient absorption and transport, and gene regulation. Results of the current study indicate, for the first time, mild-HS-induced alterations of the small-intestinal proteomes in finishing pigs.

### Stress response and defense

We reported several stress response proteins, including heat shock proteins (HSPs; HSPH1, HSPA1A, HSPA5, HSPB1 and FKBP4), and acute phase proteins (APPs; HP, AHSG, RBP4 and ALB) that were changed by HS. Many of these are heat-inducible. HSPs are a family of proteins that restore protein homeostasis and contribute to cell survival. Therefore, they have various roles, which include chaperoning, aiding the removal of damaged proteins, protein folding and transport, inhibition of cellular apoptosis, and protection cells (including intestinal epithelial cells) against thermal or oxidative stress (Horowitz & Robinson 2007). Noticeably, we found that HSPH1, HSPA1A and FKBP4 were associated and involved in the heat shock factor 1 (HSF1)-mediated heat shock response pathway. Considering these proteins functions and their interactions, overexpression of the stress response proteins may indicate their coordinating protection intestine from damage to chronic HS. These results are in agreement with recent reports in acute HS model for growing pigs (Pearce *et al*. 2013*b*), suggesting that chronic and acute HS commonly provoke heat shock response, resulting in changes in HSPs expression (Xie *et al*. 2014).

A novel and important finding of this study is that changes of APPs abundance were observed in jejunum of heat-stressed pigs. HP (haptoglobin), a known positively responding APP, increased 3.91-fold, coherent with report on dramatic increase of HP concentrations in HS pigs blood (Pearce *et al*. 2013*a*). It binds free hemoglobin and thus reduces the oxidative damage associated with hemolysis (Murata *et al*. 2004). Hp is synergistically enhanced by glucocorticoids (Baumann *et al*. 1989) and its secretion is increased during inflammatory reactions in pigs (Dobryszycka 1997). This result is consistent with increased plasma cortisol (a main glucocorticoid hormone) due to chronic HS in our previous study (Hao *et al*. 2014). In contrast, AHSG (known as fetuin), RBP4 and ALB are considered negatively responding APPs and well characterized in humans (Ombrellino *et al*. 2001, Gruys *et al*. 2005) but not as much in pigs. APPs are considered non-specific innate immune components involved in the repair of tissue damage, the restraint of microbial growth, and the restoration of homeostasis. In response to chronic heat exposure, positively- and negatively-responding APPs showed an increase and decrease in levels respectively. Moreover, inflammation or tissue injury can trigger cytokine (e.g. TNF) release by defense-oriented cells including mucosal epithelium, thereby activating the acute phase protein response (Murata *et al*. 2004). Results above indicate that chronic HS may induce inflammation or damage to mucosal epithelium?either of which would trigger HSP and APP responses to counteract the negative effects of heat exposure.

### Cell structure and motility

Cytoskeletal proteins have vital roles in the maturation, migration, and renewal of epithelial cells along the crypt–villus axis (Gordon & Hermiston 1994). In the present study, the highest representation of identified differentially expressed proteins related to the cytoskeleton reflects the effect of HS on cell structure and motility. CFL1 is a protein, which directly regulates actin dynamics and depolymerization. MYL9 plays a critical role in cell motility and contraction. Decreased intestinal integrity has been correlated with cofilin dephosphorylation and MYL9 phosphorylation respectively. Interestingly, MYL9 phosphorylation is catalyzed by MLCK and MLC phosphatase (MLCP). The catalytic activity of MLCP is attributable to PPC1B (Grassie *et al*. 2011). The up-regulation of PPC1B in the present study may accelerate the MYL9 phosphorylation, consequently resulting in increased intestinal permeability. Our current results are consistent with previous studies as both proteins were increased due to HS in the ileum (Qi *et al*. 2011, Pearce *et al*. 2014*b*).

EZR (also known as villin 2; VIL2) and VIL1, which are microvillar proteins in intestinal epithelial cells, are required for cell surface adhesion, migration, and organization (Saleh *et al*. 2009), and are thereby involved in intestinal absorption. In addition, KRT10 and KRT20 function in maintaining the cell structure as the major intermediate filament protein in the intestinal epithelia. CALD1 is a regulatory factor in the microfilament network and is thus involved in the assembly and stabilization of microfilaments in the apical portion of the intestinal epithelial cells (Ishimura *et al*. 1984). CORO1B is an actin binding protein, which regulates cell motility by coordinating actin filament turnover. Collectively, the up-regulation of CFL1 and MYL9 correlated with the down-regulation of EZR, VIL1, KRT10, KRT20, and CORO1B in heat-stressed pigs indicates orchestrated regulation of actin cytoskeletal dynamics and the negative impact of HS on intestinal integrity and TJ. This may be supported by the result of increased epithelial sloughing or atrophy of their intestinal villus.

### Glucose and energy metabolism

The gastrointestinal tract is a metabolically active organ consuming considerable amounts of energy (Cant *et al*. 1996). An important finding of this study was that HS affected expression of key enzymes involved in intestinal glucose and energy metabolism, including PGM2, MDH2, NDUFA10, NDUFS3, NDUFS, ATP5A1 and ATP5B. PGM2 and MDH2 were involved in glycolysis and the citric acid cycle, respectively, both of which were down-regulated under HS. These results indicate that HS slows down energy metabolism.

**Figure 8:**
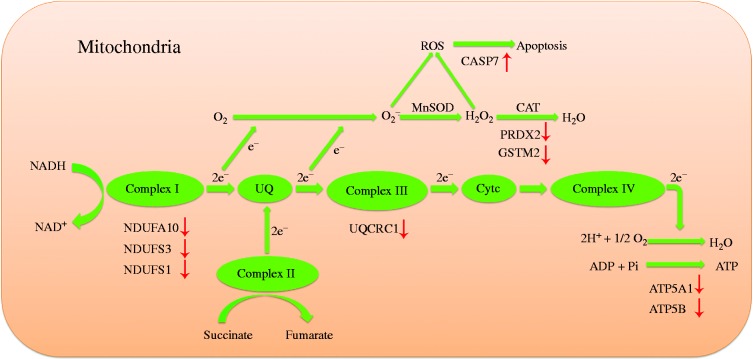
Differentially expressed proteins involved in electron transport chain (ETC) and ATP generation. The red arrows indicate up- or down-regulated proteins in response to the chronic heat stress. Protein names for the symbols used are defined in Table 4.

Another mechanism involved in energy supply is the electron transport chain (ETC) and ATP generation via oxidative phosphorylation (Fig. 8). NDUFA10, NDUFS3 and NDUFS1 are constituents of the mitochondrial electron transport chain complex I. UQCRC1 is an essential part of the mitochondrial electron transport chain complex III; all of these are responsible for pumping protons through the mitochondrial inner membrane. ATP5A1 and ATP5B, two subunits of the catalytic portion F1 of the ATP synthase complex, play a critical role in ATP generation. In the present study, heat-stressed pigs showed lower expression of ETC and ATP generation proteins. The energy derived from the passage of electrons through complexes I, III, and IV of the respiratory chain is coupled to the synthesis of ATP. Low abundance of these proteins suggests impairment in the electron transport system and ATP synthase complex, leading to compromised energy metabolism in the intestine of heat-stressed pigs. This observation is consistent with results from Weller *et al*. (2013), who revealed that genes involved in energy metabolism in muscle have reduced expression in HSed pigs. Moreover, previous research has proven that oxidative stress is related to the impairment of energy metabolism (Zaza *et al*. 2013). The electron transport chain of the mitochondria is a major source of cellular ROS. The depression of the respiratory chain by HS will lead to more formation of ROS, thus leading to cellular oxidative stress.

### Antioxidant system

Another pathway that appeared to be affected by HS is the antioxidant system. PRDX2 belongs to the ubiquitous family of peroxiredoxins that protect cellular lipids and proteins against oxidative damage generated by reactive oxygen species (ROS) (Di Quinzio *et al*. 2007). GST is crucial in the glutathione redox cycle, and catalyzes the conjugation of glutathione to a variety of electrophiles including ROS in small intestines (Aw 2005). Furthermore, a previous study has shown that HS leads to decreased glutathione in the small intestine (Pearce *et al*. 2013*a*). Glutathione is required for the activity of GST and reduced availability of glutathione may have resulted in reduced GST expression in chronic HS pig intestine. Both proteins serve as scavengers of ROS in antioxidant defense. A decrease in both GSTM2 and PRDX2 during chronic HS suggests a reduced ability of small intestine to address ROS.

### Cellular apoptosis

TRAF1, 14-3-3E (YWHAE), ERLIN2 and CASP7 participate in cellular apoptosis. TRAF1, which is a product of the NF-kB-responsive protein, can directly bind or block caspase-8 activation and prevent TNF-induced apoptosis (Chang & Tepperman 2003). The 14-3-3E, an isoform of 14-3-3 proteins, attenuates c-Abl- and ADR-induced apoptosis. Decrease in both TRAF1 and 14-3-3E may accelerate apoptosis. In addition, down-regulation of ERLIN2 suppresses degradation of inositol 1,4,5-trisphosphate receptors, leading to pathological changes in Ca^2^^+^ signaling causing cellular apoptosis (Pearce *et al*. 2007). Furthermore, CASP7 belongs to the subgroup of executioner caspases and facilitates the execution of apoptosis (Lamkanfi & Kanneganti 2010). Collectively, HS may induce intestinal epithelial cell apoptosis, which consequently compromises the intestinal epithelium barrier function.

In living cells, proteins build complex networks to fulfill these functions through protein–protein interactions, modifications, and protein regulation (Wang *et al*. 2011). The biological interaction network (BIN) clearly demonstrates that proteins related to cell structure and motility, energy metabolism and stress response and defense are the majority and account for approximately 56.6% of the BIN, which further ascertains molecular adaptive responses of the intestine during HS. These results are in line with the results of GO functional enrichment analysis.

## Conclusions

This study integrates biochemical, histological and proteomic approaches to identify the effects of chronic HS on the intestines of finishing pigs. HS results in decreased intestinal integrity and function, which might be attributed to changes of intestinal structure proteins. The down-regulation of proteins associated with the tricarboxylic acid cycle (TCA) cycle, ETC and oxidative phosphorylation suggests that chronic HS compromises energy metabolism and thus induces oxidative stress. Furthermore, in response to thermal and oxidative stress, the up-regulation of HSPs and alteration of APPs indicate formation of molecular adaptive mechanisms. The constructed BIN predicted that 28 proteins related to energy production, the cytoskeleton, stress response, and defense act as key nodes for energy metabolism, structure and immunity of the intestine. These advanced proteome data significantly expand our knowledge on effects of chronic HS on intestines.

## Figures and Tables

**Table 1:** Composition of the experimental diet

	**g/kg**
Ingredient	
Corn	662.0
Soybean meal, 42.8% CP	200.0
Wheat bran	65.0
Wheat middlings	40.0
Limestone	10.0
Dicalcium phosphate	6.0
Salt	4.0
Premix^a^	10.0
l-Lysine·HCl	3.0
Chemical composition^b^	
Digestive energy (MJ/kg)	13.39
Crude protein	157.3
Calcium	6.5
Total phosphorus	4.1
Available phosphorus	1.7
Lysine	9.2
Met+Cys	5.4

a Premix provided the following per kg of complete diet for finisher pigs: vitamin A, 2512 IU; vitamin D_3_, 1200 IU; vitamin E, 34 IU; vitamin K_3_, 1.5 mg; vitamin B_12_, 17.6 μg; riboflavin, 2.5 mg; pantothenic acid, 6.8 mg; niacin, 20.3 mg; choline chloride, 351 mg; Mn, 10 mg; Fe, 50 mg; Zn, 50 mg; Cu, 10 mg; I, 0.3 mg; Se, 0.3 mg.

b Calculated values.

**Table 2:** Effect of chronic heat stress on plasma hormone. Pigs were exposed to either thermal neutral (TN) conditions (22°C) or chronic heat stress (HS) conditions (30°C) for 3 weeks. Values represent the mean±s.d., *n*=8 pigs for each group

**Parameter**	**TN**	**HS**	***P* value**
T_3_ (ng/ml)	0.87±0.11	0.91±0.24	0.54
FT_3_ (ng/ml)	3.14±0.14	2.55±0.30	0.008^†^
T_4_ (ng/ml)	72.71±2.07	70.80±2.19	0.55
FT_4_ (pg/ml)	10.48±0.31	11.08±0.52	0.14
GH (ng/ml)	5.06±0.16	4.12±0.29	0.015*

^*^ *P*≤0.05 and ^†^ *P*≤0.01 before vs after heat stress. T_3_ (triiodothyronine); FT_3_ (free triiodothyronine); T_4_ (thyroxine); FT_4_ (free thyroxine); GH (growth hormone).

**Table 3:** The effect of constant heat stress on jejunum morphology. Pigs were exposed to either thermal neutral (TN) conditions (22 °C) or chronic heat stress (HS) conditions (30 °C) for 3 weeks. Values represent the mean±s.d., *n*=8 pigs for each group

**Parameter**	**TN**	**HS**	***P* value**
Villus height (μm)	533.37±17.20	455.37±17.42*	0.02
Crypt depth (μm)	182.12±6.29	161.62±7.78*	0.05
Villus:Crypt ratio	2.95±0.10	2.83±0.14	0.59

^*^ *P*≤0.05 before vs after heat stress.

**Table 4:** Effects of heat stress on intestinal integrity and function. Pigs were exposed to either thermal neutral (TN) conditions (22 °C) or chronic heat stress (HS) conditions (30 °C) for 3 weeks. Values represent the mean±s.d., *n*=8 pigs for each group

**Parameter**	**TN**	**HS**	***P* value**
AP (μ/g prot)	159.74±14.95	106.86±16.18*	0.008
d- lactate (μg/ml)	0.866±0.026	1.076±0.101*	0.05

*P≤0.05 before vs after heat stress. AP, alkaline phosphatase.

**Table 5:** Biochemical information about proteins differentially expressed in the small intestine of pigs exposed to chronic heat stress. *P* value, indicates the significance of up- or down-regulation of spots according to the *t*-test through ANOVA

**Spot no.** ^a^	**Protein name**	**Short name**	**Accession no.** ^b^	**Protein score** ^c^	**PM** ^d^	**TheoreticalMr(kDa)/pI** ^e^	**Fold change** ^f^	***P* value**	**Subcellular location**	**Functions**
Stress response and defense										
1	Haptoglobin (*Sus scrofa*)	HP	gi|189409353	808	16	39.01/6.51	3.91	0.0008	Secreted	Acute-phase response
5	Peptidyl-prolyl cis-trans isomerase (EC 5.2.1.8) (*Sus scrofa*)	FKBP4	gi|305855148	479	26	51.63/5.34	1.93	0.0050	Cytoskeleton, Mitochondrion, nucleus	Heat shock protein binding
6	Heat shock protein 105 kDa (*Sus scrofa*)	HSPH1	gi|148225750	640	20	97.52/5.29	1.74	0.0270	Cytoplasm	Stress response
7	Heat shock 70 kDa protein 1B (*Sus scrofa*)	HSPA1A	gi|47523308	652	24	70.34/5.60	1.46	0.0240	Cytoplasm	Stress response
32	Heat shock 70 kDa protein 5 (*Sus scrofa*)	HSPA5	gi|14916993	608	17	73.17/5.43	1.54	0.0076	Endoplasmic reticulum	Chaperone
62	S-phase kinase-associated protein 1 (*Homo sapiens*)	SKP1	gi|545856410	362	10	18.82/4.4	1.58	0.0117	Cytosol, nucleoplam	Protein modification
86	Hsp27 (*Sus scrofa*)	HSPB1	gi|55668280	557	12	22.98/6.23	1.43	0.0357	Cytoplasm, Cytoskeleton, nucleus	Stress response
24	Retinol-binding protein 4 (*Sus scrofa*)	RBP4	gi|3041715	349	8	23.39/5.41	−2.05	0.0059	Secreted	Acute-phase response
38	Serum albumin (*Sus scrofa*)	ALB	gi|833798	941	29	71.36/5.92	−1.75	0.0012	Secreted	Transport, acute-phase response
74	Alpha-2-HS-glycoprotein (*Sus scrofa*)	AHSG	gi|545865183	197	10	39.56/5.50	−1.63	0.0144	Secreted	Acute-phase response
Cell structure and motility										
2	Alpha-actinin-1 (*Sus scrofa*)	ACTN1	gi|340007404	578	38	103.11/5.33	1.61	0.0100	Cytoplasm, cytoskeleton	Tight junction
3	Peflin (*Homo sapiens*)	PEF1	gi|74761895	323	6	30.65/6.1	1.61	0.0086	Cytoplasm, membrane	Proteolysis
4	Myosin regulatory light chain, LC20 (*Sus scrofa*)	MYL9	gi|264748	542	8	19.74/4.8	1.44	0.0184	muscle myosin complex	Muscle contraction
12	PREDICTED: synemin (fragment) (*Sus scrofa*)	SYNM	gi|350578838	242	19	126.81/4.94	2.04	0.0076	Cytoskeleton	Adherens junction
13	Cofilin-1 (*Sus scrofa*)	CFL1	gi|51592135	202	8	18.79/8.16	1.81	0.0047	Cytoskeleton	Cytoskeleton organization
67	Serine/threonine-protein phosphatase PP1-beta catalytic subunit (*Homo sapiens*)	PPP1CB	gi|87621715	837	18	38.23/5.94	1.50	0.0148	Cytoplasm, Nucleus	Regulation of cell adhesion
68	Serine/threonine-protein phosphatase PP1-beta catalytic subunit (*Homo sapiens*)	PPP1CB	gi|87621715	721	16	37.96/5.94	1.53	0.0047	Cytoplasm, Nucleus	Regulation of cell adhesion
14	Non-muscle caldesmon isoform X9 (*Sus scrofa*)	CALD1	gi|545882446	169	19	62.42/6.04	−1.44	0.0430	Cytoskeleton	Muscle contraction
15	Villin 1 (*Sus scrofa*)	VIL1	gi|311273061	644	31	93.12/5.62	−1.83	0.0219	Cell projection, Cytoskeleton	Cell migration
17	Keratin, type I cytoskeletal 10 (*Homo sapiens*)	KRT10	gi|269849769	580	24	59.02/5.01	−2.22	0.0488	cellular structure	Structural molecule activity
18	Cardiac muscle alpha actin 1 (*Sus scrofa*)	ACTC1	gi|210077998	302	9	42.33/5.23	−1.61	0.0297	Cytoplasm, Cytoskeleton	Muscle contraction
28	PREDICTED: keratin, type I cytoskeletal 20 (*Sus scrofa*)	KRT20	gi|311267326	1460	34	49.09/5.38	−1.65	0.0195	cellular structure	Structural molecule activity
29	PREDICTED: keratin, type I cytoskeletal 20 (*Sus scrofa*)	KRT20	gi|311267326	285	177	49.09/5.38	−1.57	0.0145	cellular structure	Structural molecule activity
43	Keratin, type I cytoskeletal 20 (*Sus scrofa*)	KRT20	gi|311267326	1370	31	49.09/5.38	−1.68	0.0025	cellular structure	Structural molecule activity
50	Coronin-1B (*Sus scrofa*)	CORO1B	gi|350579892	379	19	54.74/6.13	−2.20	0.0177	Cytoplasm, Cytoskeleton	Cytokinesis and signal transduction
52	Ezrin (*Bos taurus*)	EZR	gi|545797521	703	35	68.83/6.06	−1.73	0.0101	cell membrance	Cell adhesion
53	High mobility group protein B1(*Homo sapiens*)	HMGB1	gi|4504425	348	12	25.05/5.62	−1.60	0.0222	Nucleus, Chromosome	Cell motility
76	Desmin (*Sus scrofa*)	DES	gi|48374063	1140	36	53.65/5.21	−1.41	0.0049	Cytoplasm	Muscle contraction
Glucose and energy metabolism										
26	Phosphoglucomutase 2 (EC 5.4.2.2) (*Sus scrofa*)	PGM2	gi|456753214	464	25	70.17/5.95	−1.92	0.0309	Cytoplasm	Glucose metabolism
31	ATP synthase subunit beta, mitochondrial (*Sus scrofa*)	ATP5B	gi|89574051	843	20	47.06/4.99	−1.64	0.0157	Mitochondrion inner membrane	ATP synthesis
33	NADH dehydrogenase (ubiquinone) 1 α subcomplex subunit 10, mitochondrial (*Sus scrofa*)	NDUFA10	gi|311273371	508	21	40.79/6.77	−1.86	0.0021	mitochondrial respiratory chain complex I	Electron transport
37	Stomatin-like protein 2, mitochondrial (*Sus scrofa*)	STOML2	gi|60415944	384	15	38.62/6.88	−1.55	0.0050	Mitochondrion inner membrane	Mitochondrial ATP synthesis coupled proton transport
39	ATP synthase subunit α, mitochondrial (*Bos taurus*)	ATP5A1	gi|114543	1390	22	56.25/5.15	−1.72	0.0011	Mitochondrion inner membrane	Electron transport
46	NADH-coenzyme Q reductase (*Sus scrofa*)	NDUFS3	gi|345090979	469	15	30.21/6.98	−1.70	0.0008	mitochondrial respiratory chain complex I	Electron transport
49	NADH-ubiquinone oxidoreductase 75 kDa subunit, mitochondrial (EC 1.6.5.3) (*Sus scrofa*)	NDUFS1	gi|311272935	877	31	80.51/5.79	−1.79	0.0469	mitochondrial respiratory chain complex I	Electron transport
51	Malate dehydrogenase (EC 1.1.1.37) (*Sus scrofa*)	MDH2	gi|164543	669	15	31.98/6.15	−1.33	0.0263	Mitochondrion	Tricarboxylic acid cycle
55	PREDICTED: ketohexokinase isoformX1 (EC 2.7.1.3) (*Sus scrofa*)	KHK	gi|311252954	784	14	33.05/5.63	1.75	0.0017	cytoplasm	Carbohydrate metabolism
56	Cytochrome b-c1 complex subunit 1,mitochondrial (*Sus scrofa*)	UQCRC1	gi|545862218	909	21	53.48/5.76	−1.52	0.0052	mitochondrial respiratory chain complex III	Electron transport
58	Glyoxylate reductase/hydroxypyruvate reductase (EC 1.1.1.79) (*Sus scrofa*)	GRHPR	gi|545805086	711	15	41.19/5.97	−1.37	0.0017	cytoplasm	Carbohydrate metabolism
Antioxidant system										
16	Glutathione S-transferase mu 2 (EC 2.5.1.18) (*Sus scrofa*)	GSTM2	gi|116047847	126	9	25.92/6.90	−3.53	0.0269	Cytoplasm	Glutathione transferase Antioxidant activity
75	Peroxiredoxin-2 (*Sus scrofa*)	PRDX2	gi|347300176	486	9	22.04/5.23	−1.75	0.0322	Cytoplasm	Antioxidant activity
Cellular proliferation and apoptosis										
8	Plasma gelsolin precursor, partial (*Sus scrofa*)	GSN	gi|164472	368	21	85.06/5.93	1.49	0.0067	Cytoskeleton, secreted	Actin filament severing; apoptotic process
70	Caspase-7 (EC 3.4.22.60) (*Sus scrofa*)	CASP7	gi|417515676	691	15	34.51/5.92	1.42	0.0012	Cytoplasm	Apoptosis
27	TNF receptor-associated protein 1 (*Sus scrofa*)	TRAF1	gi|345441801	938	32	80.09/6.58	−2.10	0.0014	Cytoplasm	Apoptosis
34	CDC37 cell division cycle 37 protein (*Sus scrofa*)	CDC37	gi|51870491	433	16	44.98/5.00	−1.57	0.0127	Cytoplasm	Cell cycle; cell division
47	Erlin-2 (*Sus scrofa*)	ERLIN2	gi|217314887	425	16	38.02/5.36	−1.81	0.0215	Endoplasmic reticulum membrane	ER-associated protein catabolic process
89	14-3-3 protein epsilon (14-3-3E) (*Homo sapiens*)	YWHAE	gi|5803225	564	18	29.33/4.63	1.97	0.0036	Cytoplasm	Apoptotic signaling pathway
Nutrient absorption and transport										
11	Inhibitor of carbonic anhydrase (Sus scrofa) (*Sus scrofa*)	ICA	gi|47523160	666	25	79.64/5.88	1.34	0.0102	Secreted	Ferric iron binding
19	Trafficking protein particle complex subunit 3 (*Su* *s scrofa*)	TRAPPC10	gi|12644285	330	8	20.46/4.88	−1.80	0.0422	Golgi apparatus	Iron Transport
21	Nascent polypeptide-associated complex subunit α-2 (*Homo sapiens*)	NACA2	gi|71152003	229	6	23.36/4.58	−2.03	0.0120	Cytoplasm, nucleus	Transport
Gene regulation										
9	M7GpppX diphosphatase (EC 3.6.1.59) (*Sus scrofa*)	DCPS	gi|60389430	667	15	38.61/5.58	1.66	0.0053	Cytoplasm, nucleus	mRNA processing
63	Ribonuclease (EC 3.1.26.4) (*Homo sapiens*)	RNASEH1	gi|212550176	313	14	34.13/5.39	1.43	0.0259	Cytoplasm	Degradation of the RNA
23	Protein mago nashi homolog (*Homo sapiens*)	MAGOHB	gi|47117708	333	14	17.21/5.74	1.80	0.0038	Nucleus	MRNA processing mRNA splicing

a Spot number as given in Fig. 1.

b Accession number according to the NCBI database.

c Statistical probability of true positive identification of the predicted protein calculated by MASCOT software from the NCBInr database.

d Number of query matched peptides.

e Theoretical Mr(kDa)/pI: molecular mass/isoelectric point of the predicted protein.

f Fold change. the protein spots showed a significant change in abundance compared to the control analyzed by* t*-test.

**Table 6:** Enriched KEGG pathway-based sets of differentially expressed proteins in the intestine of finishing pigs during chronic heat stress^a^

**Pathway name**	**Count**	**Protein**	***P* value**	***q* value**
Oxidative phosphorylation	5	ATP5A1, ATP5B, NDUFS3, NDUFA10, UQCRC1	5.9E-3	9.6E-2
Regulation of actin cytoskeleton	6	ACTN1, CFL1, EZR, MYL9, PPP1CB, GSN	6.7E-3	8.2E-2

ATP5A1, ATP synthase subunit alpha, mitochondrial; ATP5B, ATP synthase subunit beta, mitochondrial; NDUFS3, NADH-coenzyme Q reductase; NDUFA10, NADH dehydrogenase (ubiquinone) 1 alpha subcomplex subunit 10, mitochondrial; UQCRC1(cytochrome b-c1 complex subunit 1,mitochondrial; ACTN1, alpha-actinin-1; CFL1, cofilin-1, EZR, ezrin; MYL9, myosin regulatory light chain, LC20; PPP1CB, serine/threonine-protein phosphatase PP1-beta catalytic; GSN, gelsolin.

a The number of count refers to the amount of proteins involved in the extended KEGG network and pathway. *P* values are calculated according to a hypergeometric test, *q* values represent *P* values corrected for multiple testing using the false discovery rate method.
